# Sign language experience has little effect on face and biomotion perception in bimodal bilinguals

**DOI:** 10.1038/s41598-023-41636-x

**Published:** 2023-09-15

**Authors:** Jessica M. Lammert, Alexandra T. Levine, Dursa Koshkebaghi, Blake E. Butler

**Affiliations:** 1 Department of Psychology, University of Western Ontario, Western Interdisciplinary Research Building Room 6126, London, ON N6A 5C2 Canada; 2 Western Institute for Neuroscience, University of Western Ontario, London, Canada; 3 Undergraduate Neuroscience Program, University of Western Ontario, London, Canada; 4 National Centre for Audiology, University of Western Ontario, London, Canada; 5 Children’s Health Research Institute, Lawson Health Research, London, Canada

**Keywords:** Human behaviour, Sensory processing, Visual system

## Abstract

Sensory and language experience can affect brain organization and domain-general abilities. For example, D/deaf individuals show superior visual perception compared to hearing controls in several domains, including the perception of faces and peripheral motion. While these enhancements may result from sensory loss and subsequent neural plasticity, they may also reflect experience using a visual-manual language, like American Sign Language (ASL), where signers must process moving hand signs and facial cues simultaneously. In an effort to disentangle these concurrent sensory experiences, we examined how learning sign language influences visual abilities by comparing bimodal bilinguals (i.e., sign language users with typical hearing) and hearing non-signers. Bimodal bilinguals and hearing non-signers completed online psychophysical measures of face matching and biological motion discrimination. No significant group differences were observed across these two tasks, suggesting that sign language experience is insufficient to induce perceptual advantages in typical-hearing adults. However, ASL proficiency (but not years of experience or age of acquisition) was found to predict performance on the motion perception task among bimodal bilinguals. Overall, the results presented here highlight a need for more nuanced study of how linguistic environments, sensory experience, and cognitive functions impact broad perceptual processes and underlying neural correlates.

## Introduction

Experience-dependent plasticity refers to the brain’s capacity to adapt in response to sensory input and interaction with the environment, and is the primary means by which humans learn new behaviors^1^. During development, experience-dependent plasticity interacts with genetic control to shape the maturing brain^2^. However, experience-dependent plasticity continues to shape patterns of neural connectivity throughout life^3^. Reduced sound input and the introduction of visual-manual language are two striking examples of unique audio-visual experiences that are thought to influence visual perception and behavior in D/deaf individuals.

Unique patterns of neural structure and function in D/deaf individuals have been associated with changes in behavioral performance across a range of sensory and cognitive tasks^4–6^. Enhanced visual sensitivity in D/deaf individuals has been shown to be accompanied by neuroplastic changes within—at a minimum—brain regions typically associated with auditory, visual, and multisensory functions^4^. Accordingly, advantages are prevalent for functions that typically benefit from auditory-visual integration, such as perception in the peripheral visual field, where acuity is poor relative to central vision^5^.

While the case for visual functional enhancement in D/deaf individuals is compelling, it remains challenging to discern whether these changes are related to auditory deprivation, visual-manual language experience, or some combination of the two. Evidence of plasticity is often most apparent in congenitally D/deaf, or early-D/deaf individuals who experience extended periods of auditory deprivation that begin early in development^7^. However, these same individuals also often acquire sign language early in life^8^, and thus have two unique experiences that differ from hearing non-signers. This confounds interpretation since visual language experience could also affect visual perception^9^.

Signed languages consist of a complex combination of facial expressions, hand and body movements, and hand shapes that are perceived rapidly and simultaneously^9,10^. Fluent signers typically fixate on the face to perceive linguistic facial expressions and rely on peripheral vision to perceive manual gestures^10–14^. The Enhanced Exposure Hypothesis^9^ suggests this unique linguistic experience may influence general visual abilities, especially those integral to sign language communication—such as the perception of faces, complex body movements, and peripheral motion.

One of the earliest studies of face perception in sign language users showed that D/deaf signers outperformed hearing non-signers on a face matching task^15^. Interestingly, while their performance did not reach the level of D/deaf signers, hearing signers *also* outperformed non-signers, suggesting that sign language experience could account for at least part of this effect. Other studies have found that D/deaf and hearing signers perform equally well and outperform non-signers on the recognition of faces in challenging viewing conditions (e.g., when partially occluded by shadows^16^) or in conditions where speed is sacrificed for improved accuracy^17^. These studies further support the idea that improved facial recognition may result from sign language exposure rather than hearing loss per se. Interestingly, both D/deaf^16^ and hearing signers^17^ who learned sign language later in life outperform non-signers in these studies, suggesting that the effect of sign language experience is not limited to a “critical period” of development.

Studies of motion processing and target detection in the visual periphery also provide evidence of behavioral differences between sign language users and non-signers. Both D/deaf and hearing signers have demonstrated superior motion detection abilities in the right visual field compared to hearing non-signers^18,19^, who show a left visual field advantage^18^. Additionally, D/deaf signers show motion processing advantages bilaterally in the visual periphery, while hearing signers and hearing non-signers show advantages for central motion^20,21^. D/deaf signers also show unique sensitivities to hand sign motion and biological motion more generally compared to hearing non-signers^10,22^, though it is unclear to what extent these differences arise from sign language experience versus hearing loss.

In addition to behavioral studies, imaging experiments have examined how deafness and sign language experience affect patterns of brain activity evoked by visual motion. Regions of the ‘auditory’ cortex that typically respond to moving sounds have been observed to respond to visual motion in D/deaf signers, whereas hearing signers show the same pattern of activity observed in hearing non-signers^23,24^. Thus, while behavioral advantages may be associated with sign language experience irrespective of hearing status, crossmodal reorganization (i.e., neural activation to stimuli in one modality in a brain region that typically responds to a different modality) may be unique to D/deaf individuals. A similar dissociation has been observed in patterns of brain activity evoked by hand signs; auditory deprivation has been associated with increased activity in the right superior temporal cortex, while sign language experience was associated with increased activity in the bilateral superior temporal cortices, irrespective of hearing status^25^.

Apart from analyses conducted by Bosworth and colleagues^9,26^, few attempts have been made to document visual advantages associated with properties of sign language. The current study focuses on face perception and biological motion perception, which both draw on global visual processing abilities that have been shown to be enhanced in D/deaf signers^27–29^. Specifically, the goal of the current work is to determine to what degree these perceptual advantages might reflect experience with sign language rather than the lack of auditory experience.

Previous attempts to disentangle the impacts of hearing loss and visual language experience have included goups of D/deaf *non-signers*; however, these studies are often limited by small sample sizes and comorbidities^30^. Moreover, with a majority of schools and households with D/deaf children incorporating some type of sign language, and the rate of early cochlear implantation increasing^8^, D/deaf non-signers are becoming increasingly rare. Thus, the current study examines the effect of sign language proficiency by contrasting participants with typical hearing who use sign language (hereafter referred to as bimodal bilinguals^31^) and those who do not. If the perceptual advantages described previously in D/deaf signers are the result of sign language experience, bimodal bilinguals might also be expected to demonstrate superior face recognition and biological motion perception compared to controls. If, however, behavioral advantages are the result of auditory deprivation or an interaction between deprivation and visual-manual language experience, we would not expect to observe differences between these two groups.

Bimodal bilinguals have varying degrees of fluency in their two languages; some individuals may have been raised with spoken language and acquired a signed language late in life (e.g., interpreters) while others (e.g., children of D/deaf adults) may have been exposed to sign and spoken language from birth. Accordingly, while many previous studies have considered sign language experience as a dichotomous variable (e.g., signers vs. non-signers, high vs. low proficiency signers), the current study also considers whether a continuous measure of sign language proficiency might explain some of the variability in visual perceptual advantages reported in sign language users.

## Results

### Experiment 1: face perception

**Figure 1:**
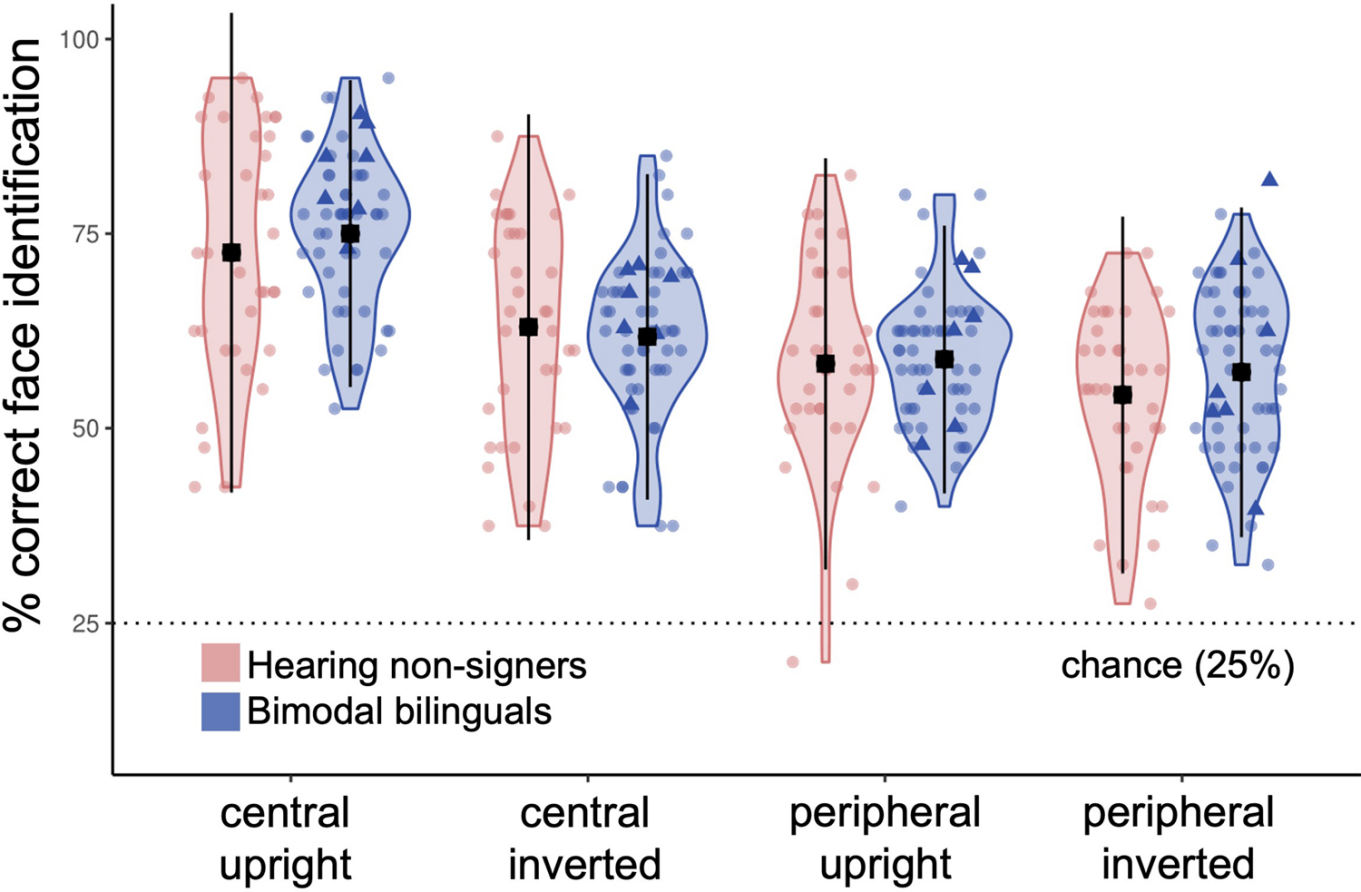
Face identification by group. Performance did not vary as a function of group in this task. Black squares represent the mean for each group in each stimulus condition. Dots show individual participant data. Dotted line shows chance level performance. Bimodal bilinguals who acquired ASL at 13-years-old or earlier are highlighted as solid triangles. Error bars show ± 2SD.

**Figure 2:**
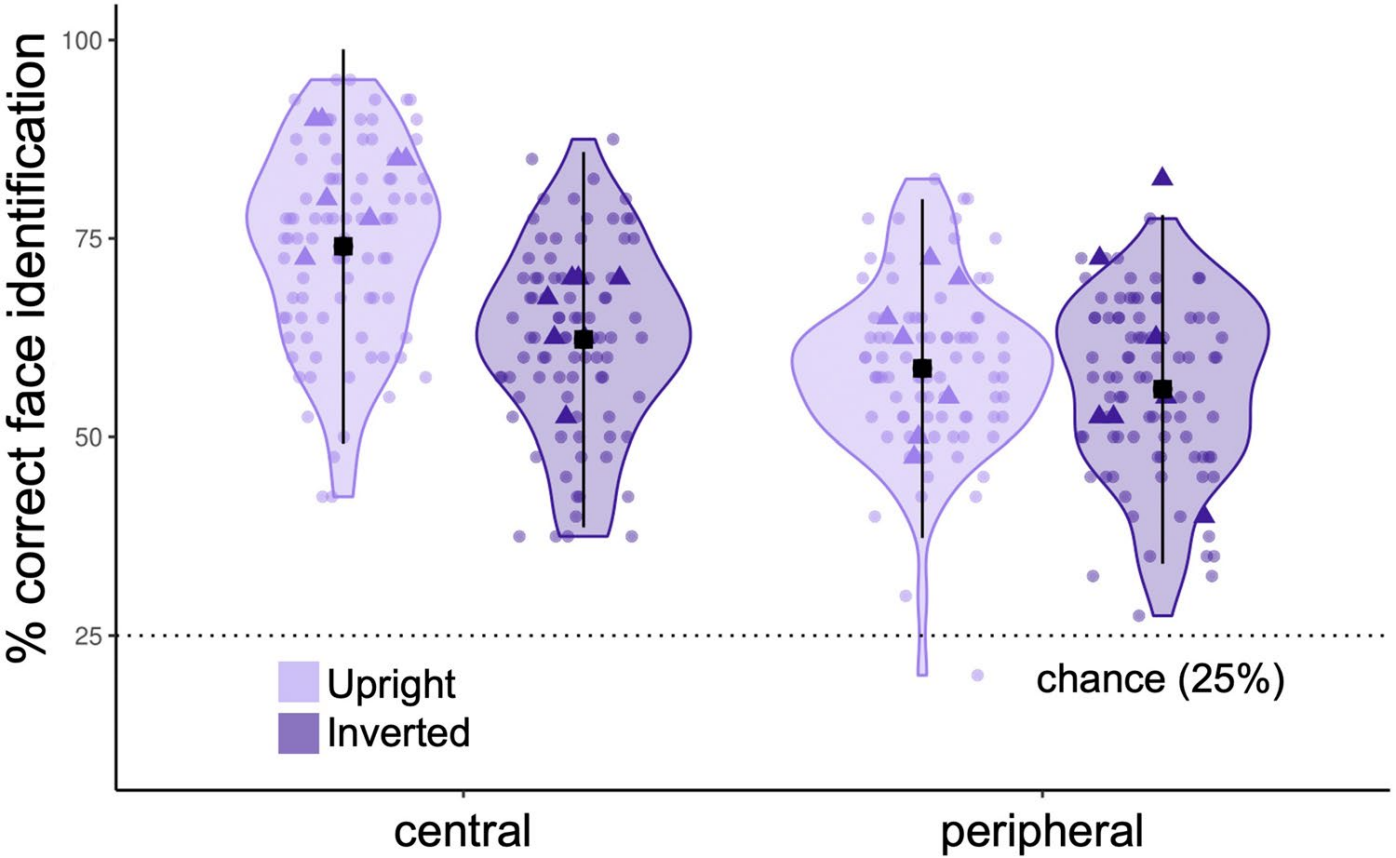
Face identification collapsed across groups. Performance was superior for upright (light) versus inverted faces (dark), and for centrally-presented (left) versus peripherally-presented faces (right). Black squares represent the mean for each group in each stimulus condition. Dots show individual participant data. Dotted line represents chance level performance. Bimodal bilinguals who acquired ASL at 13-years-old or earlier are highlighted as solid triangles. Error bars show ± 2SD.

To examine whether ASL experience directly affects the perception of human faces, bimodal bilinguals and hearing non-signers completed a delayed match-to-sample task in which they were asked to select which of four faces matched the identity of a previously viewed target face. No significant difference in face matching accuracy was observed between bimodal bilinguals and controls (F(1, 90) = 0.75, *p* = 0.39; Fig. 1). Accordingly, Bayesian analyses suggested moderate evidence for the absence of a group effect (BF_incl_ = 0.189). Across groups, performance was significantly better for faces presented in the central field compared to in the visual periphery (F(1, 90) = 141.78, *p* < 0.001, η^2^_G_ = 0.19) and for upright compared to inverted faces (F(1, 90) = 60.73, *p* < 0.001, η^2^_G_ = 0.09). However, these main effects were qualified by a significant location by orientation interaction (F(1, 90) = 26.98, *p* < 0.001, η^2^_G_ = 0.04; Fig. 2). Follow-up tests revealed superior performance in the central/upright condition compared to the central/inverted (t(88) = − 10.96, *p* < 0.001, d = 0.12), peripheral/upright (t(88) = 14.30, *p* < 0.001, d = 0.16), and peripheral/inverted (t(88) = − 16.53, *p* < 0.001, d = 0.18) conditions. Additionally, performance was better in the central/inverted condition than in the peripheral/inverted (t(88) = 5.57, *p* < 0.001, d = 0.06) or peripheral/upright (t(88) = 3.34, *p* = 0.005, d = 0.03) conditions. No other significant interactions were observed (group × orientation: F(1, 90) = 0.23, *p* = 0.63; group × location: F(1, 90) = 0.14, *p* = 0.71; group × orientation × location: F(1, 90) = 3.81, *p* = 0.054).

### ASL proficiency effects

To determine whether differences in ASL proficiency among bimodal bilinguals predicted differences in behavioral performance, signers completed the ASL comprehension test (ASL-CT^32^), and their scores were subsequently included as a covariate in face recognition analyses. Scores on the ASL-CT ranged from 40 to 90% correct for bimodal bilinguals (M = 71.05%, SD = 12.12), in accordance with normative values provided by Hauser et al.^32^ for hearing native signers (72.00%) and D/deaf non-native signers (70.50%). A factorial ANCOVA that included ASL-CT score as a between-subjects covariate revealed that controlling for proficiency did not adjust the associations between experimental conditions and performance (F(1, 55) = 3.97, *p* = 0.051). Moreover, Bayesian analyses suggested extreme evidence *against* an effect of proficiency among signers (BF_incl_ = 6.532 × 10^–19^).

### Exploratory analyses

In addition to the preregistered analyses described above, exploratory analyses were conducted to examine: (1) whether behavioral data could be predicted from measures of ASL experience; (2) whether group differences exist in reaction times in the face identification task; and (3) whether peripheral performance varied as a function of visual hemifield across groups. Neither age of acquisition (F(1, 55) = 0.12, *p* = 0.73) nor years of ASL experience (F(1, 55) = 1.53, *p* = 0.22) were significant predictors of face matching performance. Additionally, neither age of acquisition (r(55) = − 0.12, *p* = 0.39) nor years of ASL experience (r(55) = 0.12, *p* = 0.38) were significantly correlated with ASL proficiency as measured using the ASL-CT.

There were no significant differences in reaction time between groups on the face identification task (F(1, 90) = 1.53, *p* = 0.22). However, there was a significant main effect of stimulus location (F(1, 90) = 6.40, *p* = 0.01, η^2^_G_ = 0.003), with participants responding more slowly to stimuli presented in the center of the screen compared to the periphery (t(1, 88) = 3.75, *p* < 0.001, d = 0.10). Among bimodal bilinguals, neither ASL-CT score (F(1, 55) = 0.0003, *p* = 0.95), age of ASL acquisition (F(1, 55) = 3.06, *p* = 0.08), nor years of ASL experience (F(1, 55) = 1.98, *p* = 0.16) had a significant effect on face identification reaction times.

**Figure 3:**
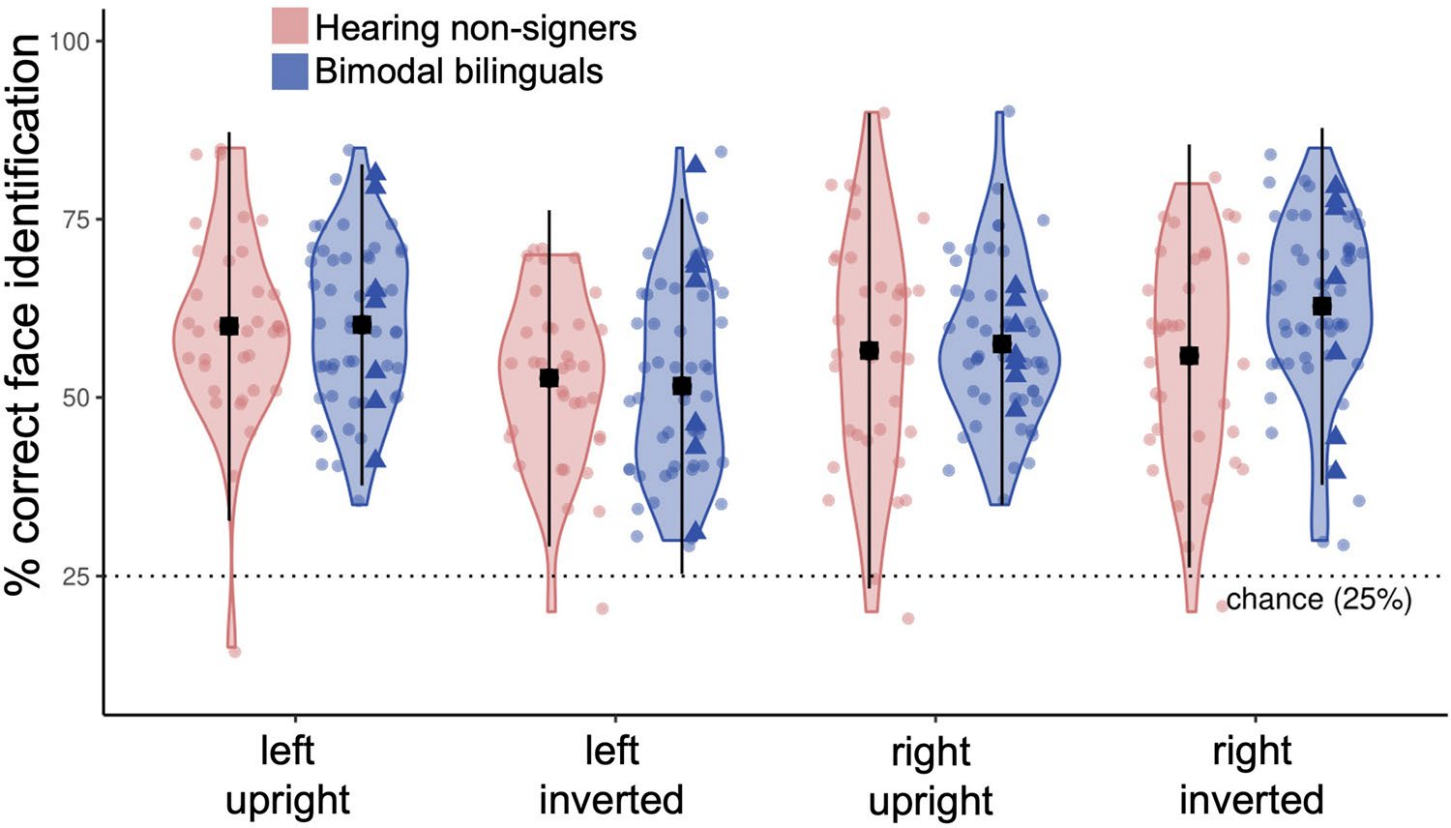
Peripheral face identification by hemifield. Bimodal bilinguals showed superior face identification for stimuli presented in the right versus the left visual hemifield. Across groups, performance was worse for inverted faces presented in the left hemifield than any other hemifield × orientation combination. Black squares represent the mean for each group in each stimulus condition. Colored dots show individual participant data. Dotted line represents chance level performance. Bimodal bilinguals who acquired ASL at 13-years-old or earlier are highlighted as solid triangles. Error bars show ± 2SD.

When visual hemifield was included as a within-subjects factor, no main effect of group (F(1, 90) = 0.98, *p* = 0.33) or hemifield (F(1, 90) = 3.82, p = 0.06) was observed. There were, however, significant group × hemifield (F(1, 90) = 4.38, *p* = 0.04, η^2^_G_ = 0.006) and orientation × hemifield (F(1, 90) = 16.76, *p* < 0.001, η^2^_G_ = 0.03) interactions. Post-hoc tests revealed that bimodal bilinguals performed better in the right hemifield compared to the left (t(1, 88) = 2.79, *p* = 0.03, d = 0.04), and that overall, participants performed worse in the left/inverted condition than in the left/upright (t(1, 88) = 4.78, *p* < 0.001, d = 0.08), right/inverted (t(1, 88) = 4.78, *p* < 0.001, d = 0.08), and right/upright (t(1, 88) = 3.04, *p* = 0.01, d = 0.05) conditions (Fig. 3). No significant interactions between visual hemifield and ASL-CT score (F(1, 55) = 1.35, *p* = 0.25), age of ASL acquisition (F(1, 55) = 1.13, *p* = 0.30), nor years of ASL experience (F(1, 55) = 0.14, *p* = 0.71) were observed among bimodal bilinguals. There were no significant effects of visual hemifield on reaction time.

### Experiment 2: biological motion perception

**Figure 4:**
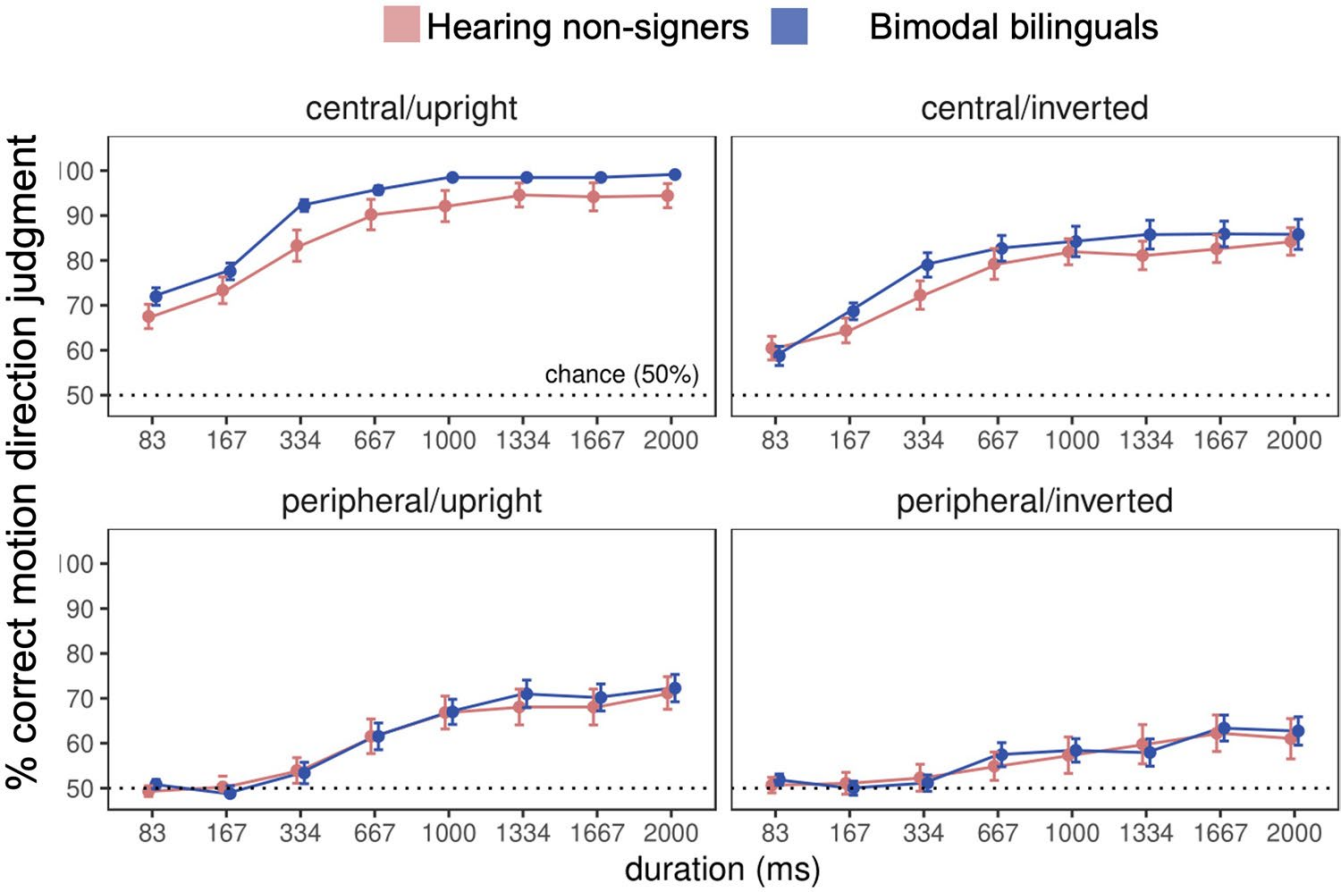
Biomotion direction discrimination by group. Performance did not differ by group across the range of orientations, locations, and durations tested. Error bars show ±SE. Dotted lines show chance level performance.

To examine whether ASL experience directly affects the perception of biological motion, bimodal bilinguals and hearing non-signers completed a direction discrimination task in which they were asked to indicate the direction in which a masked point light walker was moving. No significant difference in direction discrimination performance was observed between groups (F(1, 60) = 1.05, *p* = 0.31; Fig. 4). Moreover, Bayesian analyses suggested moderate evidence for the absence of a group effect (BF_incl_ = 0.124). Across groups, performance was better for centrally- than peripherally-presented stimuli (F(1, 60) = 256.09, *p* < 0.001, η^2^_G_ = 0.39), and for upright compared to inverted stimuli (F(1, 60) = 67.67, *p* < 0.001, η^2^_G_ = 0.07). In addition, there was a significant main effect of duration (F(1, 420) = 118.73, *p* < 0.001, η^2^_G_ = 0.20), with better performance observed at longer stimulus durations. However, these main effects were qualified by significant two-way interactions between location and orientation (F(1, 60) = 8.37, *p* = 0.005, η^2^_G_ = 0.01), location and duration (F(7, 420) = 10.82, *p* < 0.001, η^2^_G_ = 0.02), and orientation and duration (F(7, 420) = 7.84, *p* < 0.001, η^2^_G_ = 0.008), as well as a three-way interaction between location, orientation, and duration (F(7, 420) = 3.39, *p* = 0.003, η^2^_G_ = 0.003; Supplemental Fig. 1). No additional significant main effects or interactions were observed.

### ASL proficiency effects

For the bimodal bilinguals included in Experiment 2, scores on the ASL-CT ranged from 43.33 to 90.00% correct (M = 72.74%, SD = 11.18). A factorial ANCOVA revealed that including ASL proficiency as a covariate had a significant impact on the associations between experimental conditions and performance (F(1, 37) = 6.02, *p* = 0.02, η^2^_G_ = 0.04). However, this was qualified by a significant four-way interaction between ASL-CT score, location, orientation, and duration (F(1, 259) = 2.46, *p* = 0.02, η^2^_G_ = 0.004). Post-hoc tests of simple slopes (i.e., the quantification and comparison of the slopes in the effect for continuous predictors^33^) revealed that ASL-CT score had a stronger association with task performance for central than peripheral stimuli (t(257) = 6.68, *p* < 0.001, d = 0.26). However, the strength of this relationship varied considerably as a function of task parameters (see Supplemental Fig. 2). Indeed, Bayesian analyses suggested that when considered across conditions, there was extreme evidence against a generalized effect of proficiency among signers (BF_incl_ = 7.267 × 10^–211^).

### Exploratory analyses

**Figure 5:**
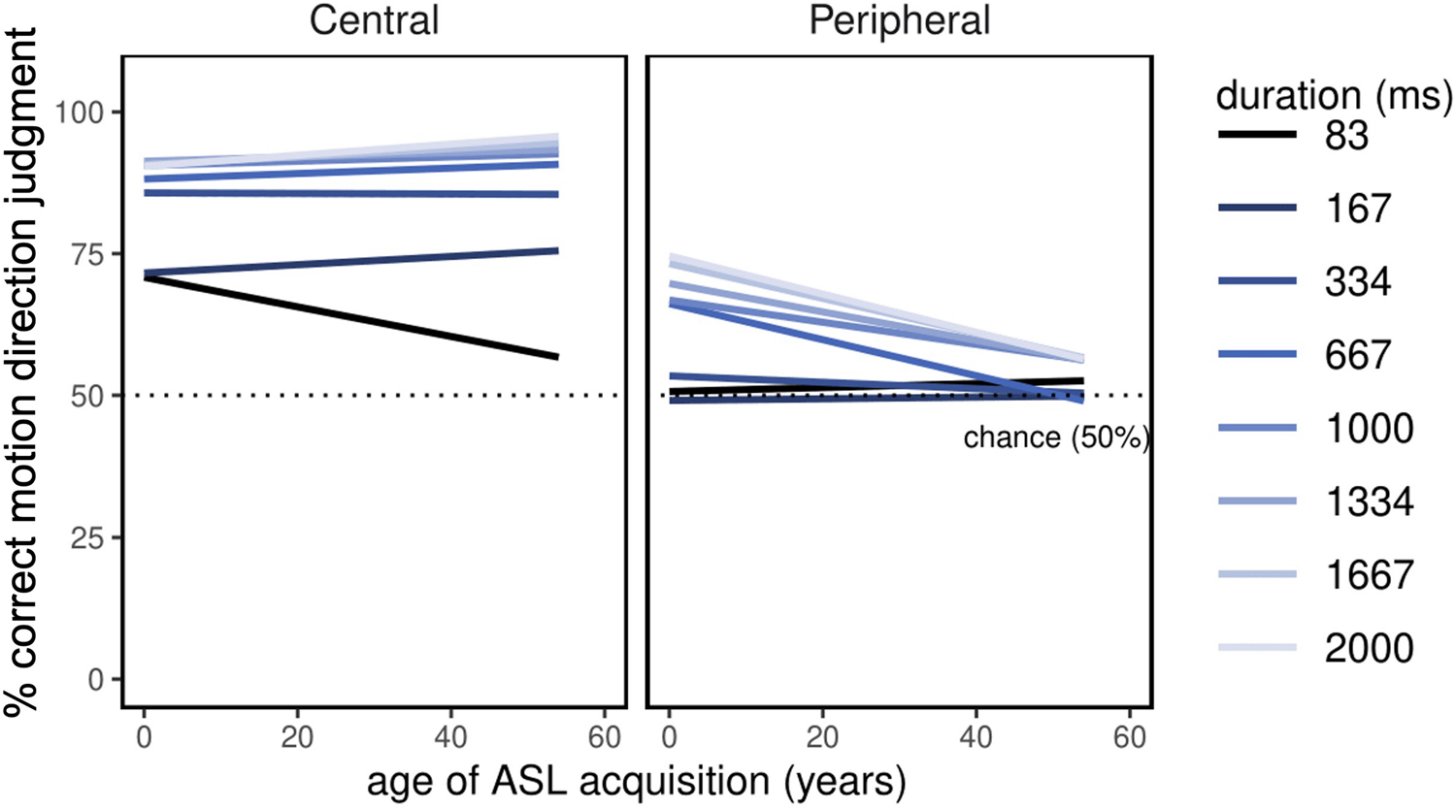
Effects of ASL proficiency on biomotion performance. Lines of best fit to the relationship between age of ASL acquisition and biomotion direction discrimination performance are shown for each location and duration combination. Early ASL acquisition predicted improved performance for brief centrally-presented walkers and for longer duration stimuli presented in the visual periphery. Dotted lines show chance level performance.

As in Experiment 1, exploratory analyses were conducted to examine effects of ASL experience, potential group differences in reaction times, and visual hemifield effects. Neither years of ASL experience (F(1, 37) = 0.06, *p* = 0.80) nor age of acquisition (F(1, 37) = 0.54, *p* = 0.47) were significant predictors of performance on the biological motion task. However, there was a significant three-way interaction between age of ASL acquisition, stimulus location, and stimulus duration (F(1, 259) = 2.77, *p* = 0.009, η^2^_G_ = 0.008). To explore this interaction, a test of simple slopes was performed, which allows one to examine how two continuous variables (e.g., ASL age of acquisition and % correct motion perception) are related at different levels of a third variable (e.g., duration), and determine whether the slopes of these regression lines differ significantly (Fig. 5). This test revealed that bimodal bilinguals who acquired ASL early outperformed late learners for very brief, centrally-presented stimuli (83 ms) and longer duration, peripherally-presented stimuli (667–2000 ms). ASL proficiency was not correlated with age of acquisition (r(37) = − 0.16, *p* = 0.32), but was related to years of ASL experience in this sample of participants (r(37) = 0.32, *p* = 0.05) however, this relationship did not survive Holm–Bonferroni correction for multiple comparisons.

**Figure 6:**
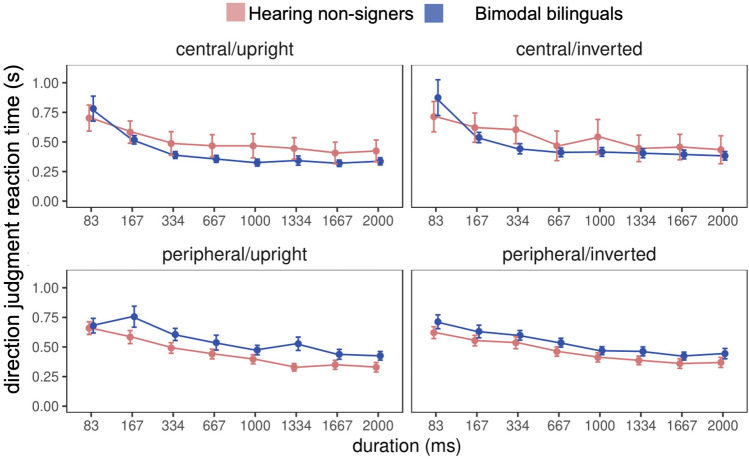
Biomotion direction discrimination reaction times by group. Reaction times did not differ by group. However, at longer durations, bimodal bilinguals (blue) were quicker to respond that hearing non-signers (red) to central stimuli, but took longer to respond to those in the visual periphery. Error bars show ±SE.

No significant effect of group on reaction times was observed (F(1, 60) = 0.04, *p* = 0.84). Across groups there was a significant main effect of stimulus duration (F(1, 420) = 64.81, *p* < 0.001, η^2^_G_ = 0.08), with participants responding more quickly as stimulus duration increased (Fig. 6). However, this main effect was qualified by a three-way interaction between group, stimulus location, and stimulus duration (F(1, 420) = 3.25, *p* = 0.002, η^2^_G_ = 0.003); at stimulus durations greater that 167 ms, bimodal bilinguals tended to respond more quickly to centrally-presented stimuli than hearing non-signers, but more slowly to stimuli presented in the visual periphery. When years of ASL experience was included as a covariate, a significant interaction between experience and stimulus location (F(1, 37) = 4.18, *p* = 0.05, η^2^_G_ = 0.005) revealed that while reaction times to centrally- and peripherally- presented stimuli were equivalent for less experienced ASL users, more experienced users responded more slowly to stimuli presented in the visual periphery (t(1, 35) = 3.35, *p* < 0.001; Supplemental Fig. 3). However, the effect size for this comparison was quite small (d = 0.003), and may be attributable to effects of aging on visual perception in our highly experienced users.

Finally, when the visual hemifield in which stimuli were presented was included as a within-subjects factor, a significant interaction between years of ASL experience and visual hemifield (F(1, 35) = 4.71, *p* = 0.04, η^2^_G_ = 0.003) was observed. While all signers showed a very slight advantage for stimuli presented in the left hemifield, this difference was found to increase somewhat with years of ASL experience (t(1, 35) = 2.59, *p* = 0.01). However, the effect size for this contrast was also quite small (d = 0.002).

## Discussion

To understand the unique effects of visual-manual language experience on visual processing, the current study compared face and biological motion perception in bimodal bilinguals (ASL users with typical hearing) and controls (individuals with typical hearing and no ASL experience). If the perceptual advantages previously demonstrated in these domains by D/deaf signers^14–25,34–39^ were the direct result of sign language experience, bimodal bilinguals might be expected to show similar advantages when compared to hearing non-signers. Moreover, the magnitude of these advantages might be predicted by an individual’s sign language proficiency.

Using a delayed match-to-sample face identification task, we observed no significant differences between bimodal bilinguals and controls, with both groups showing higher accuracy for centrally- than peripherally-presented stimuli. Additionally, there was no evidence that bimodal bilinguals’ ASL proficiency was related to their face matching performance. This contrasts with previous studies that documented enhanced face perception in bimodal bilinguals^15–17^.

There are several potential explanations for this discrepancy. In their early study, Arnold and Murray^15^ found that D/deaf signers outperformed bimodal bilinguals, who outperformed hearing non-signers on a facial memory task. Their paradigm involved showing participants multiple pairs of faces arranged in a grid and later asking them to locate image pairs from memory. The cognitive demands of this task are quite different from the current experiment, which asked participants to match the identity of a target face after a brief (500 ms) interval. However, a previous study that used the Benton Faces Test—a paradigm in which participants were asked to select a face that matches the identity of a target image from an array of six possible images—also showed that bimodal bilinguals outperform hearing non-signers^16^. Interestingly, the images presented by Bettger and colleagues^16^ contained shadows that partially obscured the subjects’ identities, suggesting that group differences in this type of task may only emerge for more difficult discriminations. Stoll and colleagues^17^ found a similar effect of sign language experience when comparing face recognition using a 2-alternative delayed match-to-sample task. However, the authors of that study note that both D/deaf and hearing signers took longer to discriminate between the familiar face and a distractor image, suggesting that the difference may lie in the trade-off between speed and accuracy across groups.

Both groups in the current study showed evidence of the “face inversion effect” in the central visual field, whereby inverted faces are more difficult to recognize than upright faces due to a disruption of global processing patterns^40^. Conversely, no inversion effects were found in the peripheral visual field. There has been some debate as to whether the peripheral visual advantages observed in D/deaf individuals result from the outward spread of central processing abilities or a redirection of central visual field attention^20^. The current findings suggest that the specialized mechanisms responsible for successful face recognition in the central visual field do not extend to the peripheral visual field in bimodal bilinguals.

For stimuli presented in the visual periphery, bimodal bilinguals showed better face recognition in the right hemifield than the left. This is consistent with previous reports of a right hemifield advantages for signers compared to non-signers^18,19^. In behavioral studies, visual hemifield advantages are thought to reflect the lateralization of associated brain activity toward the opposite hemisphere due to contralateral processing of visual stimuli^41^. Thus, the lateralized advantage demonstrated here may reflect: (1) that language processes in the brain are traditionally left-lateralized^42^; and (2) that sign language learning may selectively enhance left hemisphere/right hemifield visual processing for linguistically relevant stimuli (i.e., faces).

The behavioral results reported here are also in accordance with reports of left-lateralized brain activity in response to sign stimuli^25^ and linguistic facial expressions^43,44^ in D/deaf signers. While the bimodal bilinguals in Emmorey and McCullough’s^43^ study did not show left-lateralized activity, they also did not show the same degree of right-lateralization observed in hearing non-signers, instead presenting a pattern of activity that fell somewhere in-between (see also^45^). Overall, findings of lateralization in bimodal bilinguals are mixed; some studies have shown evidence of a right hemifield advantage for sign processing in bimodal bilinguals^46^ that is consistent with anatomical^47^ and functional^48,49^ changes observed in the left hemisphere. However, D/deaf signers show a left hemisphere/right hemifield advantage for face^46,50^ and motion processing^51,52^ that has not been observed in bimodal bilinguals. Indeed, several neuroimaging studies have revealed left-lateralized activity^23,24,43^ and volumetric asymmetry^45^ in D/deaf signers but not bimodal bilinguals.

While the current study demonstrated a right hemifield advantage for bimodal bilinguals, hemispheric dominance cannot be determined with certainty without the use of neuroimaging. Moreover, it is unclear whether potential differences are related to language at all; while left hemisphere lateralization is often attributed to the linguistic features of stimuli, the left hemisphere may be specialized for the analysis of complex motor sequences, rather than for language per se^53^. Accordingly, lateralization effects may depend on the specific measure, task, and population under study, with different functions being sensitive to different experiences during development^54^.

On the biological motion task, we also found no significant differences in accuracy between bimodal bilinguals and hearing non-signers. Both groups showed higher accuracy for upright versus inverted stimuli of all durations in the central visual field. Like faces, point-light walkers have a global perceptual organization that is subject to inversion effects^55^. Interestingly, both bimodal bilinguals and hearing non-signers also showed inversion effects for peripheral stimuli; however, these effects were only apparent for long-duration stimuli (> 667 ms), suggesting the global organization of biological motion is slow to emerge for stimuli outside of central vision.

The findings presented here are consistent with studies demonstrating that patterns of brain activity evoked by—and behavioral responses to—visual motion are generally similar between hearing signers and non-signers^20,21,56^. However, Neville and Lawson^18^ observed superior motion perception in the right peripheral visual field for bimodal bilinguals *and* D/deaf signers compared to hearing non-signers. Interestingly, only D/deaf signers exhibited stronger evoked potentials in response to peripheral motion than non-signers, suggesting potential compound effects of auditory deprivation and sign language experience in their participants^18^. Similar differences in evoked activity have been demonstrated using fMRI, where presumptive ‘auditory’ cortical regions have been observed to be responsive to visual motion in D/deaf but not hearing signers^23,24^. Future studies should include both behavioral and neuroimaging measures to better capture the unique effects of learning ASL on the brain and behavior.

ASL proficiency was shown to be a significant predictor of direction of motion discrimination, but the effect was qualified by significant interactions with stimulus location, orientation, and duration. Thus, it is possible a group difference might emerge under a particular set of stimulus parameters (i.e., for centrally-presented, inverted stimuli; Supplemental Fig. 2), but that an overall advantage for bimodal bilinguals in the current study is obscured by those conditions under which proficiency is unrelated to performance. Exploratory analysis also revealed a significant interaction between age of ASL acquisition, stimulus location, and stimulus duration; interestingly bimodal bilinguals who acquired ASL earlier in life showed better performance for a number of the longer duration, peripherally-presented stimuli used in the current study. This is consistent with findings that D/deaf and hearing signers are more accurate at discriminating moving stimuli in the periphery than hearing non-signers^18,20,37–39,56^. Taken together, these results suggest that early ASL acquisition may have an outsized effect on peripheral motion perception.

**Figure 7:**
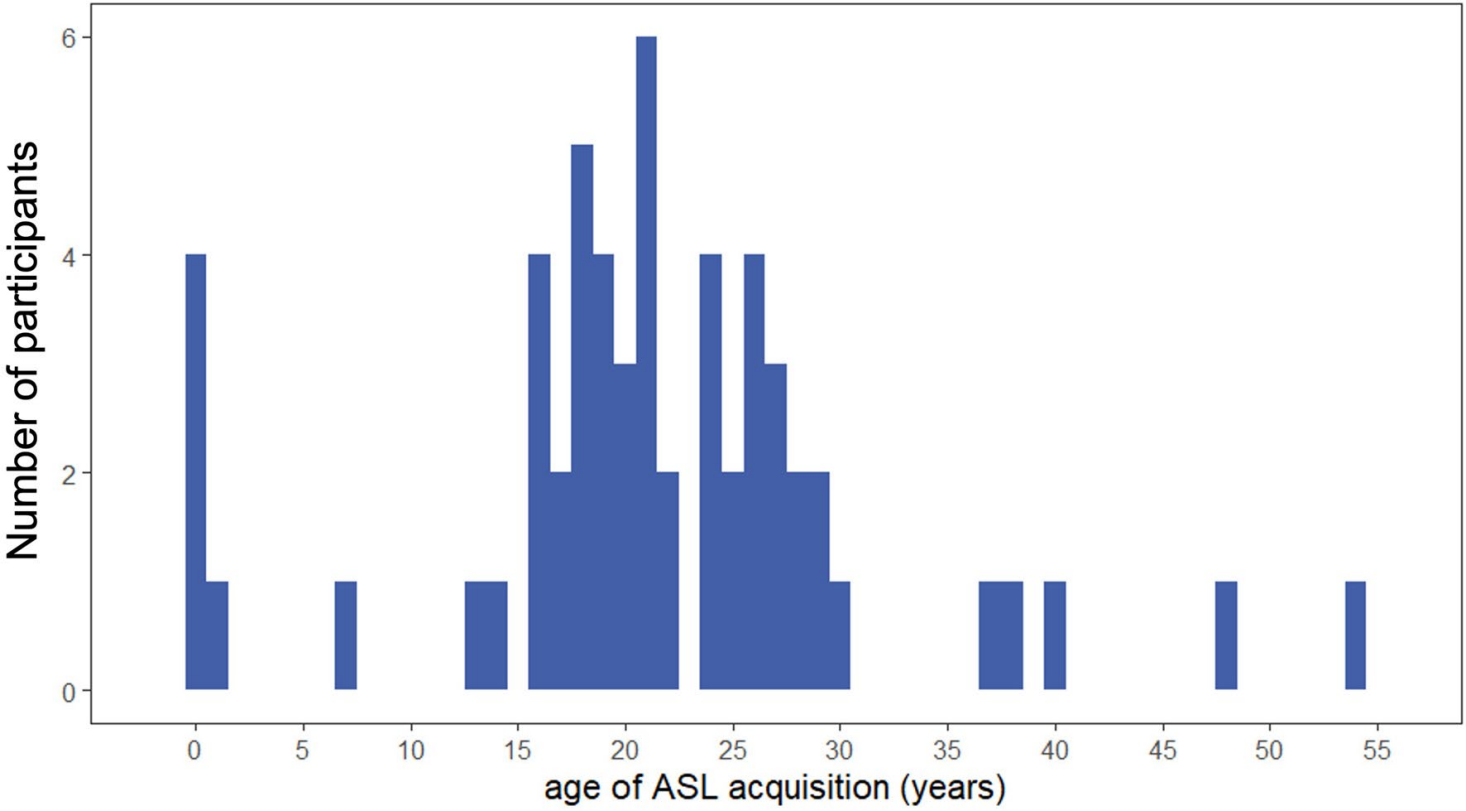
Distribution of age of ASL acquisition. Age of ASL acquisition for bimodal bilinguals in the current study ranged from 0 to 54 years of age. Median age of acquisition was 21 years.

By considering how differences in ASL proficiency might correlate with visual perception, the current study follows the general trend in the field toward more detailed assessments of language experience^57^. This includes the use of standardized measures of language proficiency and fluency to complement demographic and socio-cultural measures (e.g., age of acquisition, social diversity of language use), which may have unique effects on the brain^58^. Our group of bimodal bilinguals included a large number of ASL interpreters who, despite being proficient and frequent users of ASL, acquired sign language later in life (mean age of acquisition = 21.43 years; range = 0–54 years; Fig. 7). Accordingly, the data presented here may not reflect the patterns of behavior that might be obtained from hearing individuals with earlier ASL acquisition (e.g., children of deaf adults). Indeed, fMRI data suggest that bimodal bilinguals who learn ASL as their native language show more extensive right hemisphere activation in response to language than those who learn ASL after puberty^59^.

Interestingly, ASL proficiency was not correlated with age of acquisition *nor* years of ASL experience in the current study and was more broadly predictive of visual motion perception than either of these measures. This raises important questions regarding the extent to which factors describing language expertise (e.g., social context of language use, expressive vs. receptive skills), cognitive abilities, and motivation are being captured by measures of ‘proficiency’. Future work would benefit from efforts to quantify these factors across groups. Unfortunately, this is challenging to resolve in D/deaf signers, as many of the instruments designed to assess potential latent factors have not or cannot be translated to ASL^60^. Nevertheless, determining the unique effects of hearing loss and sign language on neural function and behavior will require a detailed understanding of language experience and the social environment.

Both face perception^34–36,43^ and motion processing^7,18–23,37–39^ have been shown to be enhanced in D/deaf individuals relative to typically-hearing controls. The current findings leave open the possibility that visual advantages in D/deaf individuals are the direct result of auditory deprivation and subsequent crossmodal reorganization^23,24,61^. Alternatively, it may be the case that visual-manual language experience affects visual perception when *combined* with auditory deprivation, resulting in a pattern of functional changes that is unique to D/deaf individuals^25^. For example, D/deaf native signers show stronger activity in response to sign language within auditory and language processing regions compared to hearing native signers (for whom auditory cortex is presumably involved primarily in the perception of acoustic stimuli^5,62^). Furthermore, more posterior regions of the superior temporal sulcus (STS) are recruited for sign language comprehension in bimodal bilinguals than in D/deaf signers^43,63^—a disparity thought to reflect the segregation of speech processing (anterior STS) and sign processing (posterior STS) that is unique to bimodal bilinguals^64^.

Given considerable evidence of enhanced visual perception in D/deaf signers, the current study explored the unique effects of sign language experience on visual perception while controlling for hearing status. Bimodal bilinguals and hearing non-signers performed similarly on measures of face and biological motion perception, suggesting that sign language experience is insufficient to induce perceptual advantages in typical-hearing adults. Despite the absence of a group difference, ASL proficiency and experience were shown to influence biomotion perception among signers for some stimuli, suggesting that sign language and other visual perceptual tasks may be related under specific conditions. It may be the case that perceptual advantages in bimodal bilinguals are limited in scope, perhaps only emerging for linguistically relevant stimuli in certain locations within the visual field. The heterogeneity of the population under study could also impact results due to the variability and complex relationship among language proficiency, age of acquisition, and frequency, duration, and context of sign language use. As one of the first studies to relate visual perception to a continuous measure of ASL proficiency, the current findings highlight the need for more detailed measures of language experience, cognition, and brain structure and function to fully understand how hearing and language experience uniquely impact perception.

## Method

The current study comprised two experiments designed to assess the impact of sign language experience on visual perception. This included assessments of face perception and biological motion perception, each designed to examine stimulus inversion effects and perceptual differences between the central and peripheral visual fields. Participants provided informed consent and completed online screening and demographics questionnaires hosted on Qualtrics (Seattle, WA) which collected information about their hearing status, vision, and language experience. Additionally, signers completed the American Sign Language Comprehension Test (ASL-CT; Hauser et al.^32^), an online measure of ASL proficiency wherein signers are presented with 30 prompts consisting of either a line-drawing, video, or signed description and are asked to select the most correct answer from four possible responses. Finally, participants completed face perception and biological motion tasks that were created in PsychoPy version 2020.1.3^65^ and hosted on Pavlovia (https://pavlovia.org).

To account for hardware variability and ensure that the size of stimuli remained consistent across screens, participants were asked to measure the height of their computer screen in centimeters and provide this value at the outset of behavioral testing. Hardware checks integrated within Pavlovia confirmed that all participants used monitors with a 60 Hz refresh rate (ensuring stimulus durations were consistent across participants). Participants were also asked to dim their lights and turn up their screen brightness, minimize distractions, place their computer on a stable surface such as a tabletop, and sit 50 cm from the screen. This study was approved by the Non-Medical Research Ethics Board at The University of Western Ontario and was undertaken in accordance with the Declaration of Helsinki. Statistical analyses and data plotting was performed using RStudio version 1.3.1073^66^ running R version 4.0.2^67^. All experimental methods and analyses across both experiments were pre-registered on the Open Science Framework (https://osf.io/6s5e3).

### Experiment 1: face perception

#### Participants

60 bimodal bilinguals (M_age_ = 37.02, SD_age_ = 11.66; 43 female, 6 male, 2 of other gender identity/non-binary) and 35 hearing non-signers (M_age_ = 32.74, SD_age_ = 11.95; 29 female, 6 male) were recruited and provided informed consent to participate. Despite reporting more than 3 years of ASL experience, three bimodal bilinguals did not exceed chance performance on the test of ASL proficiency and were excluded from subsequent analyses. The remaining 57 bimodal bilinguals reported an average of 15.59 years of ASL experience (SD = 13.20, range: 3–53 years) and mostly worked in the D/deaf community as ASL interpreters. Age of ASL acquisition ranged from 0 to 54 years of age (M = 21.43, SD = 10.32; Fig. 7). A total of seven bimodal bilinguals reported learning ASL at or before 13-years-old, including five participants who identified as children of deaf adults. All hearing non-signers self-reported no ASL experience. Furthermore, all participants self-reported normal or corrected-to-normal vision and no known hearing impairments or neurological disorders.

#### Material

**Figure 8:**
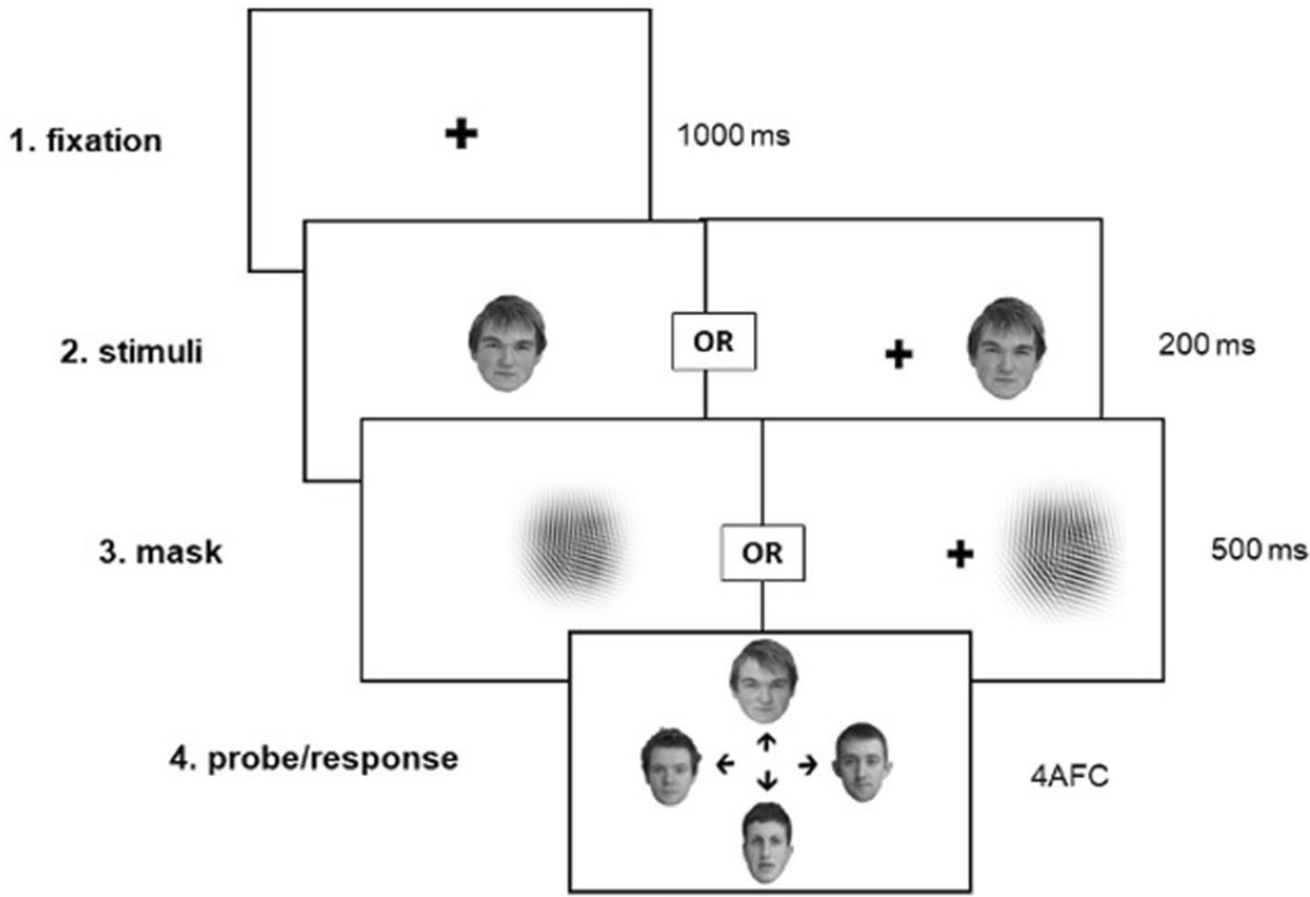
Face identification task illustration. Participants were briefly presented with a target face either at the centre of the screen or in the periphery that was followed by a visual mask. Participants were then asked to select a face from an array of four images that matched the identity of the target face. Stimuli were inverted on 50% of trials (not shown).

Whole face stimuli were selected from the Glasgow Unfamiliar Face Database^68^. Images were cropped so that only the face and hair were visible and were converted to greyscale via a custom Python script (https://osf.io/nkvhy). Each trial (48 practice, 160 experimental) consisted of the presentation of a target face, followed by an array of four faces that contained a different image of the target individual (taken on a second camera to prevent image matching) and three same-sex distractor faces previously rated as most similar to the target face^68^. Half of the trials contained male faces and half of the trials contained female faces. Each face appeared once as a target and three times as a distractor over 160 experimental rials. To compensate for cortical magnification in visual cortex (i.e., the overrepresentation of the central visual field^69^), stimuli presented in the periphery were scaled 1.25x. As a result, faces subtended 5.5° × 7.2° (width × height) when presented centrally or 6.9° × 9.0° peripherally. Each presentation of a target face was preceded by a black fixation cross at the center of the screen (0.6° squared). To disrupt afterimage effects, the target face was followed by a visual mask which consisted of four angled sinusoidal gratings subtending a visual angle of 9° squared centrally or 10.3° squared peripherally (Fig. 8). All stimuli were presented on a white background.

#### Procedure

Stimuli were blocked by experimental condition (central/upright, central/inverted, peripheral/upright, peripheral/inverted). At the outset of each block, participants received instructions that could be repeated as many times as needed. This was followed by 12 practice trials during which feedback was provided, and 40 experimental trials without feedback. During each trial, a fixation cross was presented for 1000 ms, followed by a target face shown for 200 ms, and a visual mask lasting 500 ms. The target face was presented in either upright or inverted orientation, either at the point of fixation (central) or 11° from the center of the screen (peripheral). In peripheral conditions, the fixation cross remained at the center of the screen, and the target face appeared in the left or right hemifield randomly on an equal number of trials. After viewing the target face and mask, participants were asked to identify the target face from an array of four faces as quickly and accurately as possible using their arrow keys. Identification accuracy was measured as the percentage of correct matches. The order of each condition block and the order of individual trials within each block were randomized and counterbalanced across participants via the PsychoJS trial handler in Pavlovia. Each block took approximately 5 min to complete, and participants were able to take breaks freely between blocks.

#### Planned analyses

A mixed-model ANOVA was conducted with group (bimodal bilinguals/hearing non-signers) treated as a between-subjects factor and target location (central/peripheral) and target orientation (upright/inverted) treated as within-subject factors. This ANOVA (and all analyses of variance/covariance described here) was computed using the ez package for R^70^, which provides estimates of main effects for each factor/covariate, as well as estimates of the interactions between all factors/covariates. Type III sums of squares were specified to account for the imbalance in sample sizes across groups and Tukey’s HSD test was used to examine significant main effects and interactions where appropriate.

ASL proficiency was calculated as the percentage of correct answers on the ASL-CT. To assess the influence of ASL proficiency on face perception, a separate set of analyses was performed on the subset of participants who identified as bimodal bilinguals (n = 57). This comprised a factorial ANCOVA with target location (central/peripheral) and target orientation (upright/inverted) treated as within-subject factors and ASL-CT score treated as a between-subjects covariate. Tukey’s HSD test was conducted for post-hoc analyses of categorical variables, and simple slope analyses were conducted to examine interactions with continuous variables^33^ using the emtrends function in the R packages emmeans^71^.

#### Exploratory analyses

To assess the evidence in support of group and ASL proficiency effects on face identification performance, Bayesian mixed model ANOVAs were conducted in JASP version 0.16.4^72^ with the same factors reported for the frequentist models described above.

To assess the effect of measures of ASL experience beyond proficiency, factorial ANCOVAs that included age of ASL acquisition and number of years of experience with ASL as between-subjects covariates were performed using the ez package in R^70^ on the data collected from bimodal bilinguals. Tukey’s HSD test was conducted for post-hoc analyses and simple slope analysis was conducted to examine interactions with continuous variables.

To examine whether ASL experience affected reaction time in this task, the analyses described above were replicated using reaction time as the dependent variable.

To examine whether the effect of ASL experience differed by visual hemifield, peripheral trials were divided into left- or right-side presentations and compared. An additional mixed-model ANOVA (including data from both groups) and a factorial ANCOVA (including behavioral data from bimodal bilinguals and their ASL-CT scores) were conducted as described above, with visual hemifield (left/right) included as a within-subjects factor. Tukey’s HSD test was conducted for post-hoc analyses and simple slope analysis was conducted to examine interactions with continuous variables.

### Experiment 2: biological motion perception

#### Participants

All but two of the participants who completed Experiment 1 also completed Experiment 2 (both bimodal bilinguals). However, an additional 16 bimodal bilinguals and 12 controls were excluded from analyses for failing a catch task designed to ensure fixation remained at the centre of the screen, even during long duration peripheral trials (described below). Thus, a total of 39 bimodal bilinguals (M_age_ = 35.77, SD_age_ = 10.59; 33 female, 5 male, 1 non-binary) and 23 controls (M_age_ = 29.74, SD_age_ = 10.59; 20 female, 3 male) were included in Experiment 2. The 39 bimodal bilinguals reported an average of 14.77 years of ASL experience (SD = 13.21, range: 3–53 years) and age of ASL acquisition ranged from 0 to 54 years of age (M = 21.00, SD = 11.85).

#### Material

A 90° facing point-light walker was chosen from a published set of human point-light actions^73^. The walker comprised 13 white dots positioned at the head and at each of the arm and leg joints of the figure, presented against a black background. To increase the difficulty of this task, the walker was occluded by a square mask of randomly moving dots (44 in central conditions, 22 in peripheral conditions) that were the same size and color as the dots comprising the figure. Masked walkers were presented at 60 frames/sec and completed one walk cycle (2 steps) per second. On each trial, participants were presented with a white fixation cross (0.6° square in size) at the center of the screen, followed by a masked walker for one of eight possible durations (83, 167, 333, 667, 1000, 1333, 1667, 2000 ms). To compensate for cortical magnification, stimuli in the periphery were scaled 1.25x. As a result, the masked walker subtended a visual angle of 9.7° square area in central conditions and 12° square area in peripheral conditions.

#### Procedure

**Figure 9:**
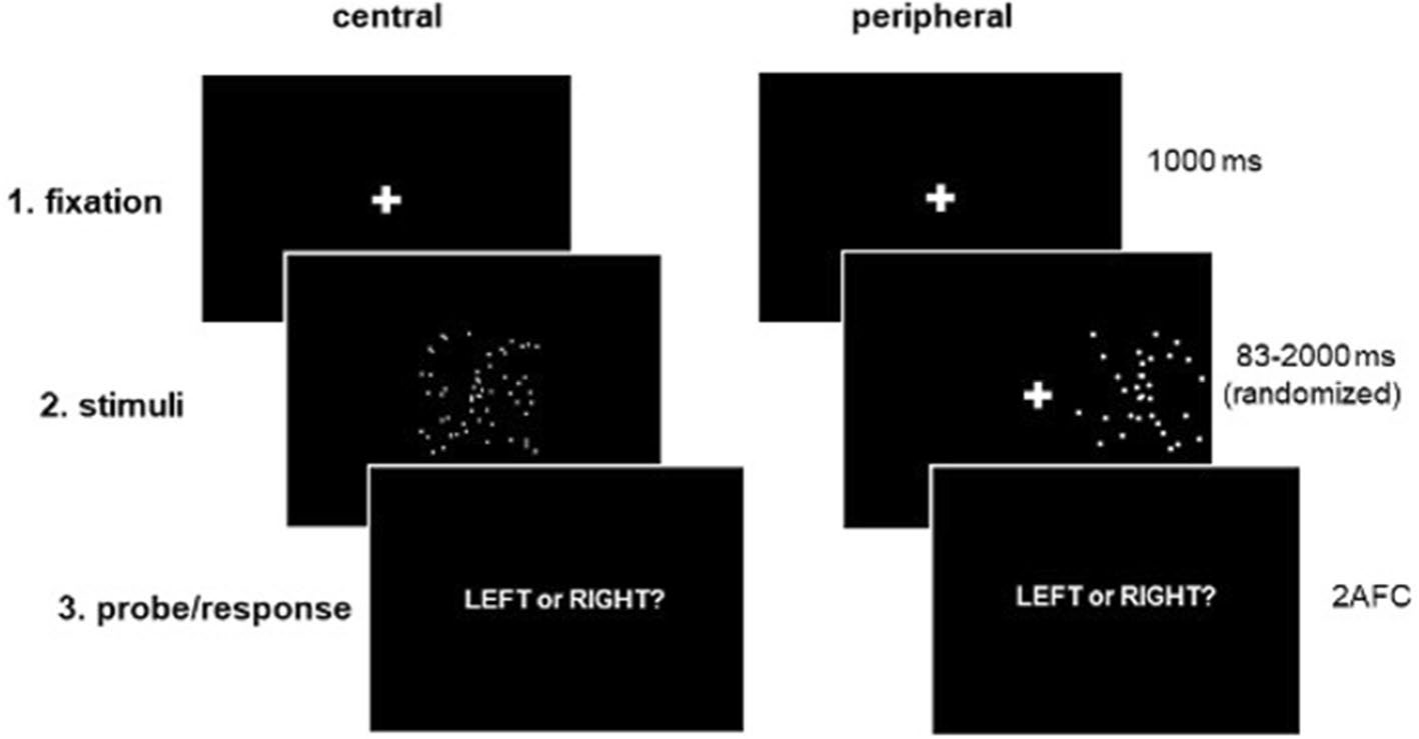
Biomotion direction discrimination task illustration. Participants saw a point light walker either at the centre of the screen or in the periphery and were asked to indicate whether the walker was moving toward the left- or right-hand side of the screen. Stimuli were inverted on 50% of trials (not show).

Stimuli were blocked by condition (central/upright, central/inverted, peripheral/upright, peripheral/inverted). At the outset of each block, participants received instructions that could be repeated as many times as needed. This was followed by 16 practice trials with feedback, and 256 experimental trials without feedback. On each trial, a fixation cross was presented for 1000 ms followed by the masked walker (Fig. 9). Practice trials in blocks of centrally-presented stimuli consisted of 8 × 1000 ms walkers and 8 × 1667 ms walkers. For peripherally-presented stimuli, practice trials included 8 × 1667 ms walkers and 8 × 2000 ms walkers. In each block, 32 experimental trials were presented at each of the 8 stimulus durations described above (83–2000 ms). On peripheral trials, stimuli appeared randomly in the left or right hemifield an equal number of times and the fixation cross remained at the center of the screen for the total duration of the trial (1083–3000 ms).

The walker appeared to be walking toward the left- or right-hand side of the screen an equal number of times across all trials. After viewing the masked walker, participants were immediately asked to indicate the direction of motion (leftward or rightward) as quickly and accurately as possible using the “right” and “left” arrow keys. Direction of motion perception accuracy was measured as the percentage of correct responses at each stimulus duration.

In central conditions, the starting position of the walker was randomly displaced by 0.5° visual angle from center to prevent participants from recognizing the walker simply from the starting position on the screen. Twenty-four randomly presented catch trials were included in peripheral blocks to ensure participants remained fixated at the center of the screen. On these trials, the fixation cross changed from white to grey for 300 ms at either 500 ms, 1000 ms, or 1500 ms post-stimulus-onset, and participants were instructed to ignore the targets and respond using the “up” arrow key. Four catch trials were presented with feedback during the practice period at the outset of peripheral blocks, and participants were reminded to maintain their gaze at the fixation throughout the experiment. Participants who subsequently failed to respond correctly to at least 17/24 (70%) catch trials were excluded from analyses. The order of experimental blocks, stimulus durations, and walker direction were all randomized across participants, and each block took approximately 15 min to complete. Eight break points were provided within each block, and participants could take additional breaks between blocks.

#### Planned analyses

A mixed-model ANOVA was conducted with group (bimodal bilinguals/controls) as a between-subjects factor and stimulus location (central/peripheral), stimulus orientation (upright/inverted), and stimulus duration (8 levels ranging 83–2000 ms) as within-subject factors. Tukey’s HSD test was conducted to further examine significant main effects and interactions as appropriate.

To assess the influence of ASL proficiency on biological motion perception, a separate set of analyses were performed on the subset of participants who identified as bimodal bilinguals (n = 39). A factorial ANCOVA was conducted with stimulus location (central/peripheral), stimulus orientation (upright/inverted), and stimulus duration (8 levels ranging 83–2000 ms) treated as within-subject factors and ASL-CT score treated as a between-subjects covariate. Tukey’s HSD test was conducted for post-hoc analyses and simple slope analysis was conducted to examine interactions with continuous variables.

#### Exploratory Analyses

Exploratory analyses examining (1) the strength of the evidence in support of group and proficiency effects, (2) potential effects of the age of ASL acquisition and years of ASL experience; (3) potential differences in reaction times across groups and conditions; and (4) potential differences across the left and right visual hemifields were conducted as described for Experiment 1 above.

### Supplementary Information

Supplementary Figures.

## Data Availability

All experimental data, as well as task and analysis scripts have been made available via the Open Science Framework website at https://osf.io/nm8j5/.
