# Supplementary material for: Sign language experience has little effect on face and biomotion perception in bimodal bilinguals
Source: Sci Rep. 2023 Sep 15;13:15328. doi: 10.1038/s41598-023-41636-x (PMC10504335; doi:10.1038/s41598-023-41636-x)
Supplement: Supplementary file 1 — Supplementary Figures. [file 41598_2023_41636_MOESM1_ESM.docx]

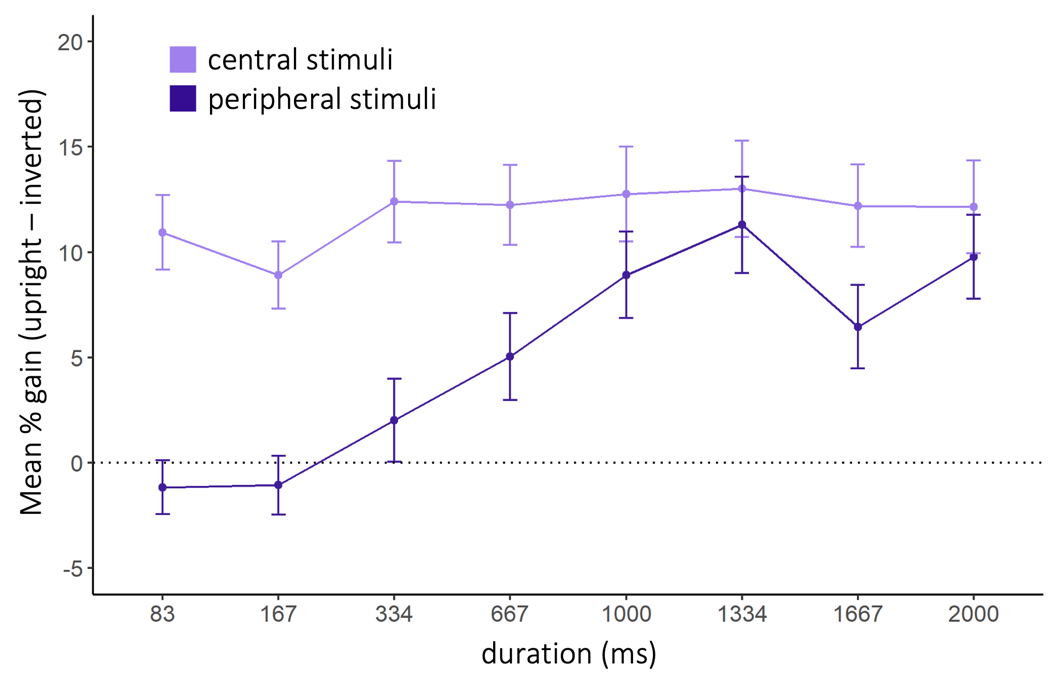


**Supplemental Fig. 1. Inversion effects for biomotion direction discrimination at different stimulus durations.** An analysis of biomotion directional judgements revealed a significant three-way interaction between stimulus orientation, location, and duration. Here, inversion effects (i.e., improvements in performance for upright vs. inverted faces) are shown for each stimulus duration presented (data collapsed across groups). While the inversion effect was relatively consistent across durations for centrally-presented stimuli (light), similar effects emerged only for longer duration stimuli when presented in the periphery (dark). Error bars show +/- 1 SE.


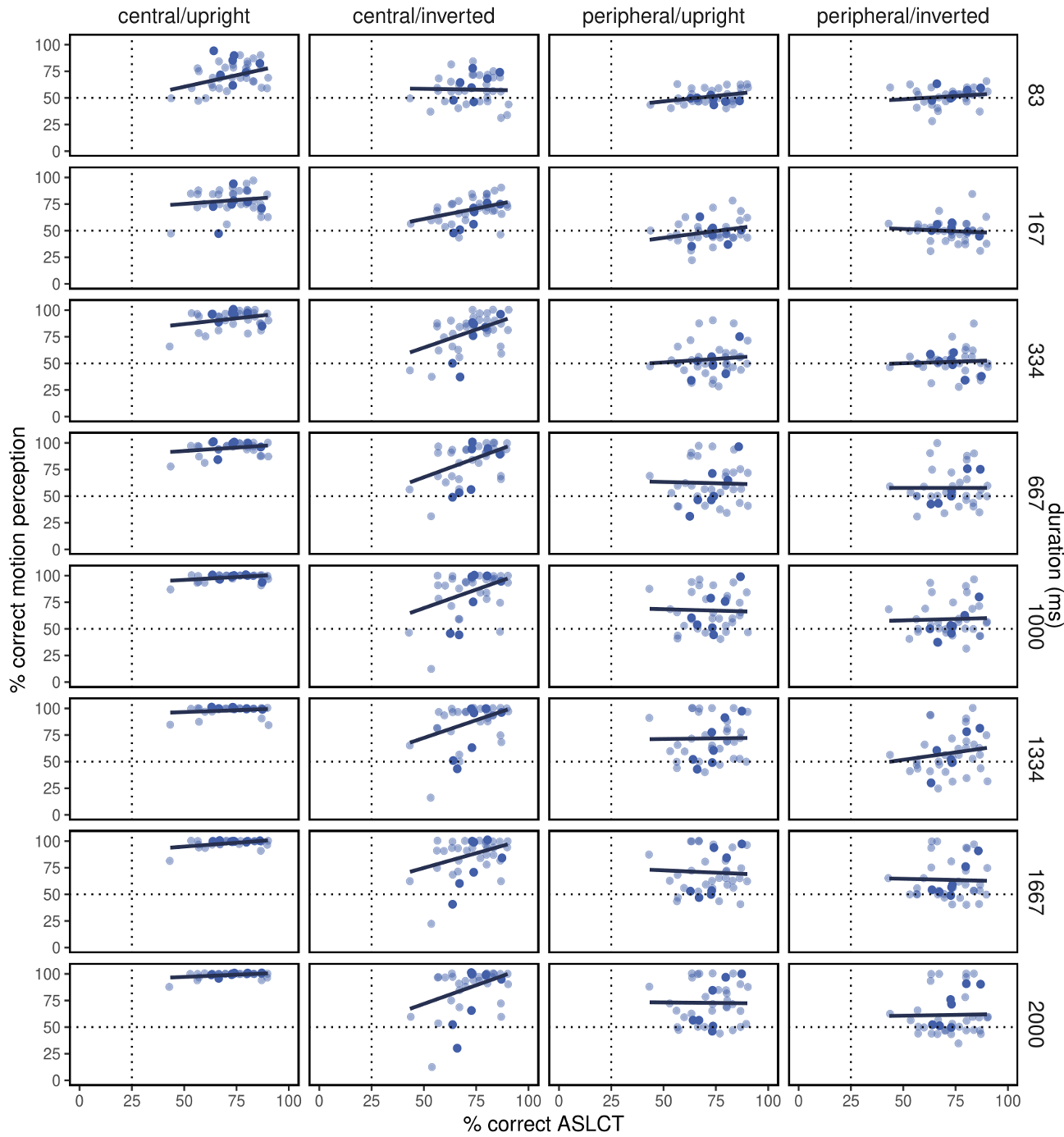


**Supplemental Fig. 2. Effects of ASL proficiency on biomotion performance.** When included as a covariate of interest, sign language proficiency was found to be predictive of biomotion direction perception accuracy for a subset of the stimulus conditions tested. Vertical dotted lines show chance performance on the ASL-CT (25%). Horizontal dotted lines show chance performance on the biomotion task (50%). Solid lines represent the best fit to the data. Dots show individual participant scores, with signers who acquired ASL at 13-years-old or younger highlighted in a darker color.


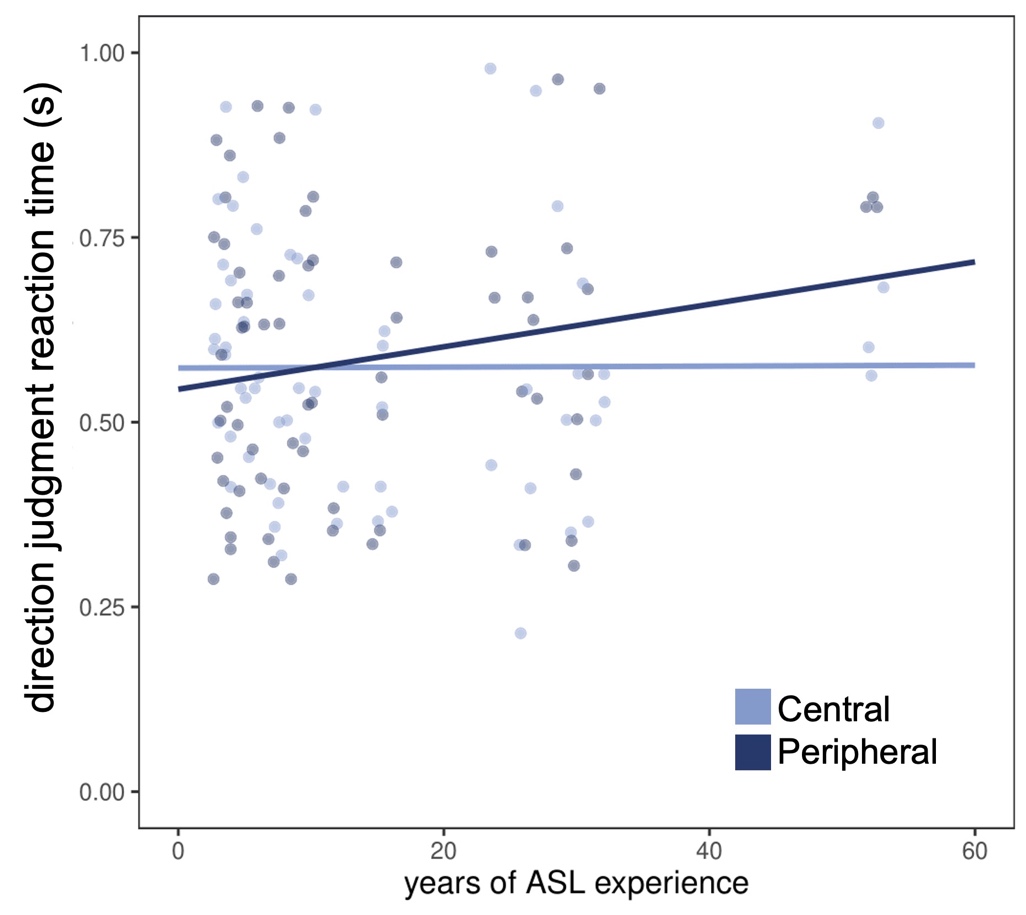


**Supplemental Fig. 3. Effect of years of ASL experience on biomotion reaction time.** When included as a covariate of interest, years of ASL experience was found to be predictive of biomotion perception reaction times in an interaction with stimulus location. Dots show individual participants scores for each location.
